# Sites of blood collection and topical disinfectants associated with contaminated cultures: An ambidirectional cohort study

**DOI:** 10.1002/jgf2.667

**Published:** 2023-12-17

**Authors:** Koshi Ota, Daisuke Nishioka, Emi Hamada, Kanna Ota, Yuriko Shibata, Akira Takasu

**Affiliations:** ^1^ Department of Emergency Medicine Osaka Medical and Pharmaceutical University Takatsuki City Japan; ^2^ Research and Development Center Osaka Medical and Pharmaceutical University Takatsuki City Japan; ^3^ Department of Nursing Osaka Medical and Pharmaceutical University Hospital Takatsuki City Japan; ^4^ Department of Clinical Laboratory Osaka Medical and Pharmaceutical University Hospital Takatsuki City Japan

**Keywords:** blood culture, contamination, site, topical disinfectant

## Abstract

**Background:**

We aimed to determine whether puncture sites for blood sampling and topical disinfectants are associated with rates of contaminated blood cultures in the emergency department (ED) of a single institution.

**Methods:**

This single‐center, ambidirectional cohort study of 548 consecutive patients ≥20 years of age was performed in the ED of a university hospital in Japan over a 13‐month period. Pairs of blood samples were collected for aerobic and anaerobic cultures from patients in the ED. Physicians selected puncture sites and topical disinfectants according to their personal preference.

**Results:**

Potential contamination was identified in 110 of the 548 patients (20.1%). One hundred fourteen (20.8%) patients showed true‐positive results for bacteremia, and 324 (59.1%) patients showed true‐negative results. Multivariate analysis revealed more frequent contamination when puncture sites were disinfected with povidone‐iodine (PVI) than with alcohol/chlorhexidine (ACHX) (adjusted risk difference, 19.1%; 95% confidence interval [CI]), 15.7–22.6; *p* < 0.001). In terms of blood collection sites, femoral and central venous (CV) catheter with PVI disinfection showed more frequent contamination than venous sites with ACHX (adjusted risk differences: 26.6%, 95% CI 21.3–31.9, *p* < 0.001 and 41.1%, 95% CI 22.2–59.9, *p* < 0.001, respectively).

**Conclusion:**

Rates of contaminated blood cultures were significantly higher when blood was collected from the CV catheter or femoral sites with PVI as the topical disinfectant.

## 1 INTRODUCTION

Blood cultures are one of the most important tests to detect life‐threatening bacteremia, which is associated with high mortality rates. Accurate diagnosis of cultured blood samples plays a crucial role in appropriate treatment. Contaminated blood cultures can result in unnecessary antibiotic use, unnecessarily long hospital stays, increased healthcare costs, and, most importantly, an increased risk of antimicrobial resistance.^1^, ^2^ Several strategies have been proposed to reduce blood culture contamination. However, a meta‐analysis has reported that only two methods can achieve this: sampling from a separate venipuncture site or a well‐trained phlebotomy team.^3^ Although topical 1.0% alcohol/chlorhexidine gluconate (ACHX) reduces blood culture contamination more effectively than 10% aqueous povidone‐iodine (PVI),^4^, ^5^ both agents are routinely applied at our institution as topical disinfectants before blood sampling. Rates of false‐positive culture are significantly reduced when blood is sampled from various venipuncture sites compared with intravenous or central venous catheters.^6^, ^7^ Physicians at our institution may sample blood from various sites, such as intravenous and central venous catheters, as well as femoral arteries and veins, according to personal preference.

The emergency department (ED) is the frontline to investigate unwell patients, with blood culture contamination rates above the recommended 3%.^3^, ^8^ A previous study for a period of 6 months revealed that blood sampled from femoral arteries or veins and using PVI was associated with a significantly higher contamination rate in our ED.^9^ The study showed that rates of contamination of femoral sites, peripheral sites, and central venous catheter were 73.6%, 16.1%, and 7.7%, respectively.^9^

We thus aimed to investigate and validate in greater detail which puncture sites for blood sampling and which topical disinfectants are associated with blood culture contamination in patients and samples from a single ED over a longer period.

## 2 METHODS

### 2.1 Study design

This single‐center, ambidirectional cohort study proceeded at the ED of a university hospital in Japan between August 1, 2018, and August 31, 2019. The hospital is an 882‐bed university teaching hospital with 8000 adults presenting to the ED annually. Strengthening the Reporting of Observational studies in Epidemiology (STROBE) guidelines were used to design and report the results from this study.^10^ The institutional review board at our institution approved the study protocol (675(2476)) and waived the need to obtain written informed consent.

### 2.2 Patients

This study included 548 consecutive patients ≥20 years of age from whom blood was sampled in the ED. Inclusion criteria were as follows: age ≥20 years and at least one pair of blood cultures collected in the ED. Patients were excluded if all blood samples were collected elsewhere. If one pair of blood samples was collected at our ED and another was collected elsewhere or no second pair was collected, then only the pair collected at our ED was analyzed. One or more of the following comorbidities of patients were recorded: malignancy, diabetes mellitus, hypertension, prior stroke, dementia, chronic renal insufficiency, liver cirrhosis, and coronary artery disease.^11^, ^12^, ^13^, ^14^

Death data were also analyzed in February 2021.

### 2.3 Blood cultures

Nurses and other medical staff at our institution are not permitted to collect blood for cultures. Only physicians, typically first‐ or second‐year residents, are permitted to collect blood samples for blood culture in the ED.

Blood (14–20 mL) from peripheral veins or arteries was sampled for aerobic and anaerobic cultures (7–10 mL each) in BacT/Alert FA Plus and FN Plus resin bottles (bioMérieux Inc., Durham, NC, USA). The use of sterile gloves was mandatory at our institution when the physician took blood culture samples. Physicians selected the topical disinfectants such as ACHX, PVI, and alcohol available in the ED, according to their personal preferences. A blood culture was considered contaminated if one or more of the following organisms were identified in one of the two blood cultures: coagulase‐negative staphylococci (CoNS), *Propionibacterium acnes*, *Micrococci*, *Corynebacteria*, *Bacillus* species other than *Bacillus anthracis*, or *Clostridium perfringens*.^7^, ^15^, ^16^ Viridans group streptococci are regarded as contaminants based on the described criteria,^7^, ^15^ but are not considered contaminants in our institute. Polymicrobial cultures showing a mixture of contaminant and true pathogens were regarded as contaminated.^14^ A culture was defined as “negative” when bacterial growth was absent. The source of infection was identified based on a chart review with other cultures such as sputum, urine, and ascites or with other modalities including ultrasonography, X‐ray, computed tomography (CT), and magnetic resonance imaging (MRI). Details are described in Table 1.

**TABLE 1:** Characteristics of patients with blood cultures in the emergency department.

Characteristics of patients	True bacteremia	True negative	Contamination	*p*
	*n* = 114	*n* = 324	*n* = 110	
Mean age, years (SD)	72.6 (11.8)	67.3 (16.7)	74.3 (11.4)	<0.001
Male sex, *n* (%)	64 (56.1)	189 (58.3)	75 (68.2)	0.126
Major comorbidities, *n* (%)				
Malignancy	50 (43.9)	124 (38.4)	40 (36.4)	0.473
Diabetes mellitus	26 (22.8)	58 (18.0)	25 (22.7)	0.383
Hypertension	41 (36.0)	80 (24.8)	43 (39.1)	0.005
Previous stroke	10 (8.8)	24 (7.4)	9 (8.2)	0.888
Chronic renal insufficiency	14 (12.3)	29 (9.0)	7 (6.4)	0.302
Liver cirrhosis	5 (4.4)	9 (2.8)	1 (0.9)	0.288^a^
Coronary artery diseases	10 (8.8)	19 (5.9)	9 (8.2)	0.488
Dementia	7 (6.1)	21 (6.5)	13 (11.8)	0.153
Quick SOFA, *n* (%)				0.087
0	37 (32.5)	125 (38.6)	40 (36.4)	
1	33 (29.0)	114 (35.2)	41 (37.3)	
2	35 (30.7)	77 (23.8)	23 (20.9)	
3	9 (7.9)	8 (2.5)	6 (5.5)	
Origin of infection, *n* (%)				
Central nervous system	1 (0.9)	3 (0.9)	0 (0.0)	0.823^a^
Pulmonary	12 (10.5)	94 (29.0)	41 (37.3)	<0.001
Cardiovascular system	6 (5.3)	2 (0.6)	0 (0.0)	0.003^a^
Abdomen	19 (16.7)	35 (10.8)	12 (10.9)	0.234
Urinary tract	47 (41.2)	53 (16.4)	13 (11.8)	<0.001
Skin	6 (5.3)	9 (2.8)	9 (8.2)	0.283
Other or unknown	26 (22.8)	137 (42.3)	39 (35.5)	0.001
Death	37 (32.5)	99 (30.6)	35 (31.8)	0.92
Death within 30 days	11 (9.7)	34 (10.5)	5 (4.6)	0.169

Abbreviations: SD, standard deviation; SOFA, sequential organ failure assessment.

^a^ Fisher's exact test was performed because of small numbers of patients in several cells. Other comparisons were analyzed using the Chi‐squared test and one‐way analysis of variance. Origin of infection means the cause of infection according to a medical chart review with several cultures and with diagnostic modalities. The central nervous system includes meningitis, encephalitis, and brain abscess. The pulmonary system includes pneumonia, bronchitis, pleuritis, and upper respiratory infection. The cardiovascular system includes endocarditis and pericarditis. The abdomen includes cholangitis, gastroenteritis, cancer of the gastrointestinal tract, hepatitis, cholecystitis, appendicitis, and pancreatitis. The urinary tract includes pyelonephritis, cystitis, and prostatitis. Skin includes decubitus, cellulitis, impetigo, and erysipelas. Other or unknown includes febrile neutropenia and cases where the source of infection cannot be identified.

### 2.4 Statistical analysis

Categorical variables are described as frequencies and percentages (%), and continuous variables are shown as mean with standard deviation (SD). Data were compared using one‐way analysis of variance, the Chi‐squared test, and Fisher's exact test, as appropriate. Differences in risk and robust 95% confidence intervals (CIs) of contamination according to sites and topical disinfectants were estimated using uni‐ and multivariate analyses with modified least‐squares regression and a robust standard error estimator.^17^, ^18^ The same patients were considered to be a random effect in the above model. Age, sex, and doctors' experience were adjusted as confounders in multivariate analyses. Because blood can be sampled from few sites, we also included blood in five categories: CV catheter, blood sampled from a newly inserted central venous (CV) catheter; femoral, blood sampled from the femoral artery or vein; other, blood sampled from a newly inserted arterial catheter and implanted port; venous, blood sampled from venipuncture without catheter insertion; and venous catheter, blood sampled from a newly inserted venous catheter. Because we did not have many topical disinfectants to assess, we included only PVI, ACHX, and other types (alcohol and benzalkonium) in analyses. We did not impute missing values. For all statistical investigations, values of *p* < 0.05 were taken as significant. All analyses were performed using STATA version 16.1 (Stata Corp., College Station, TX, USA).

## 3 RESULTS

### 3.1 Baseline characteristics

We analyzed data from 548 patients and 1066 pairs of blood cultures between August 1, 2018, and August 31, 2019. A total of 138 (12.9%) potential contaminants from 110 patients (20.1%) were found in blood cultures, the most common of which was *Staphylococcus epidermidis* in 55 of the 138 blood samples (39.9%), followed by *Staphylococcus hominis* in 25 samples (18.1%). Four patients (2.9%) had a mixture of true bacteremia and contaminating isolates. We identified true bacteremia in 114 patients (20.8%) and 224 blood samples (21.0%), with *Escherichia coli* as the most prevalent microorganism, in 59 of the 224 blood samples (26.3%). Cultured blood samples from 324 patients (59.1%) and 704 blood samples (66.0%) were identified as true negative. The most common source of infection in 47 patients (41.2%) with true bacteremia was urinary tract infection, whereas a pulmonary source was the most prevalent in 41 patients (37.3%) with contaminated cultures and 94 patients (29.0%) with true‐negative cultures. These two sources significantly differed among the three groups (pulmonary and urinary tract; both *p* < 0.001). Only one pair of blood samples was cultured from 31 patients. Neither total number of deaths nor death within 30 days differed significantly between the three groups. Tables 1 and 2 show other baseline characteristics of the three groups of patients and blood cultures. Status of physicians collecting blood samples for blood culture in the ED is shown in Table 3.

**TABLE 2:** Characteristics of blood cultures in the emergency department.

Characteristics of blood cultures	True bacteremia	True negative	Contamination	*p*
Site, *n* (%)	*n* = 224	*n* = 704	*n* = 138	
CV catheter	7 (3.1)	11 (1.7)	11 (8.0)	<0.001
Venous catheter	11 (4.9)	45 (6.4)	1 (0.7)	0.010^a^
Other	9 (4.0)	20 (2.8)	2 (1.5)	0.385^a^
Venous	75 (33.5)	305 (43.3)	14 (10.1)	<0.001
Femoral	122 (54.5)	322 (45.7)	110 (79.7)	<0.001
Disinfectants, *n* (%)				
PVI	136 (60.7)	419 (59.5)	129 (93.5)	<0.001
ACHX	84 (37.5)	261 (37.1)	8 (5.8)	<0.001
Other types	4 (1.8)	26 (3.7)	1 (0.7)	0.104^a^
One pair	3 (1.3)	24 (3.4)	4 (2.9)	0.317^a^

Abbreviations: ACHX, 1.0% alcohol/chlorhexidine gluconate; CV catheter, blood culture sample from a newly inserted central venous catheter; Femoral, blood culture sample from a femoral artery or vein; Other types, alcohol and benzalkonium; Other (site), a newly inserted arterial catheter or implanted port; PVI, 10% aqueous povidone‐iodine; Venous, venipuncture without catheter insertion; venous catheter, blood culture sample from a newly inserted venous catheter.

^a^ Fisher's exact test was performed because of the small numbers of patients in several cells. Other comparisons were analyzed using the Chi‐squared test.

**TABLE 3:** Status of physicians collecting blood samples for blood culture in the emergency department.

	True bacteremia (*n* = 224)	True negative (*n* = 704)	Contamination (*n* = 138)
First‐year residents	71 (31.7)	224 (31.8)	50 (36.2)
Second‐year residents	40 (17.9)	144 (20.5)	29 (21.0)
Physicians experienced less than or equal to 10 years	97 (43.3)	277 (39.3)	49 (35.5)
Physicians experienced more than 10 years	16 (7.1)	59 (8.4)	10 (7.2)

*Note*: First‐year residents mean first‐year trainees (postgraduate year 1 [PGY1]). Second‐year residents mean second‐year trainees (postgraduate year 2 [PGY2]). Physicians experienced less than or equal to 10 years mean that physicians who had finished their postgraduate clinical training for 2 years, and their experience was more than 2 years and less than or equal to 10 years. Physicians experienced more than 10 years mean that physicians who had finished their residency program, and their experience was more than 10 years.

### 3.2 Sites and topical disinfectants

Femoral arteries and veins tended to be sampled in most patients in all three groups, but at different ratios (Tables 1 and 2). Over 80% of blood samples from patients with contaminants were collected from femoral sites (mostly the femoral artery), whereas <50% of blood samples from patients with negative cultures were collected from these sites. Blood sampled from a newly inserted central venous catheter conferred the greatest risk of contamination when taken as an independent factor. However, the number of samples was small (*n* = 30).

Topical disinfectants also differed significantly among groups (Table 2). Disinfection with PVI was performed for sites in >90% of patients with contaminated blood cultures, compared with <65% among patients in the other two groups. Other topical disinfectants comprising alcohol or benzalkonium are also shown in Table 2.

### 3.3 Proportion of contamination by sites and topical disinfectants

With reference to ACHX, univariate analysis using modified least‐squares regression associated PVI and other types with contamination (proportions of contamination associated with PVI, other type or ACHX: 21.4% vs. 3.2% vs. 2.3%; risk difference, 19.1%; 95% CI 15.7–22.6, *p* < 0.001; 1.0%, 95% CI −5.5 to 7.4, *p* = 0.77, respectively). With reference to blood collected from venous catheters, univariate analysis using modified least‐squares regression associated femoral sites, CV catheter, other (including newly inserted arterial catheters as well as implanted ports), and venous venipuncture sites with contamination (proportions of contamination associated with femoral sites, CV catheter, other, and venous venipuncture sites: 20.6%, 40.0%, 9.7% and 6.3%, respectively; risk difference, 18.8% (95% CI 14.0–23.6; *p* < 0.001), 38.2% (95% CI 20.3–56.2; *p* < 0.001), 9.7% (95% CI −3.1 to 18.9; *p* = 0.157), and 4.6% (95% CI 0.4–8.8; *p* = 0.032), respectively (Table 4).

**TABLE 4:** Univariate analysis using modified least‐squares regression.

	Contaminants (%)	Risk difference	95% CI	*p*
Topical disinfectants				
PVI	21.4	19.1	15.7 to 22.6	<0.001
Other types	3.2	1.0	−5.5 to 7.4	0.77
ACHX	2.3	Reference		
Blood sampling sites				
CV catheter	40.0	38.2	20.3 to 56.2	<0.001
Other	9.7	7.9	−3.1 to 18.9	0.157
Femoral	20.6	18.8	14.0 to 23.6	<0.001
Venous	6.3	4.6	0.4 to 8.8	0.032
Venous catheter	1.8	Reference		

Abbreviations: ACHX, 1.0% alcohol/chlorhexidine gluconate; CI, confidence interval; CV catheter, blood culture sample from a newly inserted central venous catheter; Femoral, blood culture sample from femoral artery or vein; Other, blood culture sample from a newly inserted arterial catheter and implanted port; Other types, alcohol and benzalkonium; PVI, 10% aqueous povidone‐iodine; Venous, venipuncture without catheter insertion; Venous catheter, blood culture sample from a newly inserted venous catheter.

Using multivariate analysis, we assessed associations between sites and topical disinfectants with contamination. With reference to venous venipuncture sites and ACHX, PVI and femoral sites and PVI and CV catheter were significantly associated with contamination (adjusted risk differences: 26.6% [95% CI 21.3–31.9; *p* < 0.001] and 41.1% [95% CI 22.2–59.9; *p* < 0.001], respectively). ACHX and venous catheter and other‐type disinfectant and CV catheter were also associated with significantly decreased contamination, but the numbers of these combinations were 25 and 1, respectively (Table 5). With reference to first‐year residents, second‐year residents, physicians experienced less than or equal to10 years, and physicians experienced more than 10 years were not significantly associated with contamination (Table S1).

**TABLE 5:** Multivariate analysis using modified least‐squares regression.

	Risk difference	95% confidence interval	*p*
Explanatory variable			
Disinfectants and blood sampling sites			
ACHX and venous	Reference		
ACHX and other	−0.2	−3.4 to 3.0	0.904
ACHX and femoral	0.4	−3.1 to 3.9	0.815
ACHX and CV catheter	−2.4	−6.4 to 1.6	0.239
ACHX and venous catheter	−2.7	−5.2 to −0.2	0.035
PVI and other	12.7	−3.2 to 28.6	0.118
PVI and femoral	26.6	21.3 to 31.9	<0.001
PVI and venous	7.0	2.7 to 11.4	0.002
PVI and CV catheter	41.1	22.2 to 59.9	<0.001
PVI and venous catheter	0.6	−7.0 to 8.2	0.88
Other types and other	−2.3	−5.7 to 1.1	0.194
Other types and femoral	4.1	−9.4 to 17.6	0.55
Other types and venous	−1.3	−4.8 to 2.2	0.457
Other types and CV catheter	−4.7	−7.9 to −1.5	0.004
Other types and venous catheter	−0.8	−5.7 to 4.0	0.736
Covariates			
Male (reference female)	1.2	−2.9 to 5.3	0.56
Age (per 10 years)	1.5	0.3 to 2.7	0.012

Abbreviations: ACHX, 1.0% alcohol/chlorhexidine gluconate; CI, confidence interval; CV catheter, blood culture sample from a newly inserted central venous catheter; Femoral, blood culture sample from the femoral artery or vein; Other, blood culture sample from a newly inserted arterial catheter and implanted port; Other types, alcohol and benzalkonium; PVI, 10% aqueous povidone‐iodine; Venous, venipuncture without catheter insertion; Venous catheter, blood culture sample from a newly inserted venous catheter.

## 4 DISCUSSION

This single‐center, ambidirectional cohort study found that blood samples collected from femoral areas and CV catheter insertion at the internal jugular vein disinfected with PVI were significantly associated with contaminated blood cultures in the ED. As revealed by the previous study, the most common source of infection among patients with true bacteremia was urinary tract infection, and most such patients had pyelonephritis.^9^ In contrast, the most prevalent source of infection among patients with contamination was pulmonary disease, with most such patients having aspiration pneumonia, again similar to the previous study.^9^ Mortality rates were not significantly different among contamination, true‐negative results and true bacteremia.

We also found that three combinations (femoral puncture site with PVI, CV catheter insertion at internal jugular vein with PVI, and venous venipuncture with PVI) comprised independent risk factors for blood culture contamination. A previous similar study showed that the femoral site is unsuitable for blood cultures,^9^, ^19^ but this investigation suggested that the femoral site may be acceptable only if ACHX is used. Other disinfectants (alcohol or benzalkonium or both) might be effective to prevent contamination when blood samples were collected from the femoral site. However, the combination of the femoral site and ACHX should not be selected because this combination was only seen for 14 samples, and the data were thus insufficient for reliable statistical analysis. Furthermore, as femoral sites are colonized more often than other sites,^20^ they are associated with catheter‐related bloodstream infection.^21^ Internal jugular sites were mostly chosen for CV catheter insertion at our hospital using PVI, and this site has been confirmed to show a higher risk of contamination.^9^, ^19^, ^20^, ^21^, ^22^ In addition, the CV catheter is not suitable as a route for blood culture sampling compared with arterial lines or direct peripheral venipuncture.^23^, ^24^ The contamination rate in the present study was highest (40.0%) for the 30 blood samples collected from central catheters newly inserted into the internal jugular vein in the ED. However, this number was relatively low. Only one case of CV catheter insertion using alcohol was not contaminated, and this combination was significantly less frequent than the combination of ACHX with venous venipuncture without catheter insertion. However, we should not use this combination because only one case showed this combination, and the data were thus insufficient for reliable statistical analysis. ACHX has been recommended when a CV catheter is inserted.^25^ Handling and disinfection of access points, such as needleless connectors, antiseptic barrier caps, and catheter hubs, are complicated with CV catheters. Hence, the number of manipulations per blood collection through a newly inserted CV catheter is higher than that with arterial catheters or venous catheters.^24^, ^26^

Several reports have described associations between blood samples collected from newly inserted peripheral venous catheter and blood culture contamination.^6^, ^27^ According to those reports, collecting blood culture specimens through a newly inserted peripheral venous catheter increases the risk of contamination compared with venipuncture. Our results suggested that blood culture from a newly inserted peripheral venous catheter might be the best procedure to prevent blood culture contamination in the ED, and a review article also emphasized the need to balance the practical advantages of this method against the risk of increased contamination.^28^

Several reports have described associations between topical disinfectants and blood culture contamination.^29^, ^30^ A meta‐analysis found that blood culture contamination was more significantly reduced by ACHX than by PVI.^4^ However, physicians have tended to disinfect puncture sites with PVI more frequently than with ACHX at our hospital, for various reasons. First, physicians and ED staff are more familiar with PVI than with ACHX because it has been applied as a skin disinfectant for many years. Second, residents and medical students are not educated about blood culture procedures while at university.

Pneumonia was the most common illness among patients with contaminated and true‐negative blood cultures. Several studies of patients with community‐acquired pneumonia have found that blood cultures provide little diagnostic benefit.^31^, ^32^, ^33^ One reason for the very high contamination rate at our hospital was the high proportion of blood samples collected from femoral areas disinfected with PVI. Blood samples should be cultured from selected immunocompromised patients, those with complicating urinary tract infection under antibiotic therapy at the time of blood collection, and patients with suspected endocarditis.^32^, ^34^

All physicians are mandated to complete 2 years of accredited postgraduate clinical training in Japan with rotations in major clinical specialties, including emergency medicine.^35^ Our result and previous study showed that experience status of the physician performing sample collection was not associated with contaminated blood cultures in the ED (Table 3; Table S1).^22^ Initially, sample collection by residents was considered a likely risk factor for blood culture contamination; however, no such significant association with blood culture contamination was identified. Residents and physicians who chose femoral area with PVI were associated with contaminated blood cultures in the ED.

Several strategies have been advocated to reduce rates of blood culture contamination. Sampling from various venipuncture sites and reliance on a well‐trained phlebotomy team can reduce these rates.^3^ Furthermore, switching from PVI to ACHX or other topical disinfectants and informational intervention and feedback might reduce rates of blood culture contamination, even when physicians conduct phlebotomies.^36^, ^37^

This study shows several limitations. Our patient cohort was not large, and some parameters could not be conclusively determined. Nevertheless, specific injection sites and PVI were associated with significantly increased contamination rates. Some physicians were aware of this study proceeding within the ED and might have been more attentive when collecting blood than they might have been in wards. Physicians could select their preferred topical disinfectant for blood sampling, which also have represented a confounder, because many studies have associated contamination more with PVI than with ACHX. Blood collection sites prepared using the same disinfectant should be compared under the same conditions.

## 5 CONCLUSIONS

This single‐center, ambidirectional cohort study found that femoral puncture sites and PVI as well as CV catheter insertion and PVI were independent risk factors for blood culture contamination. Newly inserted venous catheters with ACHX should be used to obtain blood samples to reduce culture contamination.

## AUTHOR CONTRIBUTIONS

Koshi Ota and Daisuke Nishioka designed the study and wrote the initial draft of the manuscript. Kanna Ota, Daisuke Nishioka, Emi Hamada, Yuriko Shibatad, and Akira Takasu contributed to the analysis and interpretation of data and assisted in the preparation of the manuscript. All authors contributed to data collection and interpretation and critically reviewed the manuscript. All authors approved the final version of the manuscript and agree to be accountable for all aspects of the work in ensuring that questions related to the accuracy or integrity of any part of the work are appropriately investigated and resolved.

## CONFLICT OF INTEREST STATEMENT

The authors have stated explicitly that there are no conflicts of interest in connection with this article.

## ETHICS APPROVAL STATEMENT

The Institutional Review Board (IRB) at Osaka Medical College approved the study protocol (No. 675(2476)).

## PATIENT CONSENT STATEMENT

Patient consent statement was waived the need to obtain written, informed consent.

## CLINICAL TRIAL REGISTRATION

The study protocol (No. 675(2476)) at Osaka Medical College (OsakaMedical and Pharmaceutical University).

## Supporting information


Table S1.

## References

[1] Dempsey C , Skoglund E , Muldrew KL , Garey KW . Economic health care costs of blood culture contamination: a systematic review. Am J Infect Control. 2019;47:963–967. 10.1016/j.ajic.2018.12.020 30795840

[2] Alahmadi YM , Aldeyab MA , McElnay JC , Scott MG , Darwish Elhajji FW , Magee FA , et al. Clinical and economic impact of contaminated blood cultures within the hospital setting. J Hosp Infect. 2011;77(3):233–236. 10.1016/j.jhin.2010.09.033 21216032

[3] Snyder SR , Favoretto AM , Baetz RA , Derzon JH , Madison BM , Mass D , et al. Effectiveness of practices to reduce blood culture contamination: a Laboratory Medicine Best Practices systematic review and meta‐analysis. Clin Biochem. 2012;45(13–14):999–1011. 10.1016/j.clinbiochem.2012.06.007 22709932 PMC4518453

[4] Caldeira D , David C , Sampaio C . Skin antiseptics in venous puncture‐site disinfection for prevention of blood culture contamination: systematic review with meta‐analysis. J Hosp Infect. 2011;77(3):223–232. 10.1016/j.jhin.2010.10.015 21194791

[5] Marlowe L , Mistry RD , Coffin S , Leckerman KH , McGowan KL , Dai D , et al. Blood culture contamination rates after skin antisepsis with chlorhexidine gluconate versus povidone‐iodine in a pediatric emergency department. Infect Control Hosp Epidemiol. 2010;31(2):171–176. 10.1086/650201 20025532

[6] Norberg A , Christopher NC , Ramundo ML , Bower JR , Berman SA . Contamination rates of blood cultures obtained by dedicated phlebotomy vs intravenous catheter. JAMA. 2003;289(6):726–729. http://www.ncbi.nlm.nih.gov/pubmed/12585951 12585951 10.1001/jama.289.6.726

[7] Hall KK , Lyman JA . Updated review of blood culture contamination. Clin Microbiol Rev. 2006;19(4):788–802. 10.1128/CMR.00062-05 17041144 PMC1592696

[8] Bool M , Barton MJ , Zimmerman PA . Blood culture contamination in the emergency department: an integrative review of strategies to prevent blood culture contamination. Australas Emerg Care. 2020;23(3):157–165. 10.1016/j.auec.2020.02.004 32253130

[9] Ota K , Oba K , Fukui K , Ito Y , Hamada E , Mori N , et al. Sites of blood collection and topical antiseptics associated with contaminated cultures: prospective observational study. Sci Rep. 2021;11(1):6211. 10.1038/s41598-021-85614-7 33737624 PMC7973780

[10] von Elm E , Altman DG , Egger M , Pocock SJ , Gøtzsche PC , Vandenbroucke JP . The strengthening the reporting of observational studies in epidemiology (STROBE) statement: guidelines for reporting observational studies. Int J Surg. 2014;12(12):1495–1499. 10.1016/j.ijsu.2014.07.013 25046131

[11] Schellevis FG , van der Velden J , van de Lisdonk E , van Eijk JT , van Weel C . Comorbidity of chronic diseases in general practice. J Clin Epidemiol. 1993;46(5):469–473. http://www.ncbi.nlm.nih.gov/pubmed/8501473 8501473 10.1016/0895-4356(93)90024-u

[12] García‐Olmos L , Salvador CH , Alberquilla Á , Lora D , Carmona M , García‐Sagredo P , et al. Comorbidity patterns in patients with chronic diseases in general practice. PLoS One. 2012;7(2):e32141. 10.1371/journal.pone.0032141 22359665 PMC3281110

[13] Prados‐Torres A , Calderón‐Larrañaga A , Hancco‐Saavedra J , Poblador‐Plou B , van den Akker M . Multimorbidity patterns: a systematic review. J Clin Epidemiol. 2014;67(3):254–266. 10.1016/j.jclinepi.2013.09.021 24472295

[14] Lee C‐C , Lee N‐Y , Chuang M‐C , Chen P‐L , Chang C‐M , Ko W‐C . The impact of overcrowding on the bacterial contamination of blood cultures in the ED. Am J Emerg Med. 2012;30(6):839–845. 10.1016/j.ajem.2011.05.026 22169577

[15] Bekeris LG , Tworek JA , Walsh MK , Valenstein PN . Trends in blood culture contamination: a College of American Pathologists Q‐Tracks study of 356 institutions. Arch Pathol Lab Med. 2005;129(10):1222–1225. 10.1043/1543-2165(2005)129[1222:TIBCCA]2.0.CO;2 16196507

[16] Lee CC , Lin WJ , Shih HI , Wu CJ , Chen PL , Lee HC , et al. Clinical significance of potential contaminants in blood cultures among patients in a medical center. J Microbiol Immunol Infect. 2007;40(5):438–444. http://www.ncbi.nlm.nih.gov/pubmed/17932605 17932605

[17] Hagiwara Y , Fukuda M , Matsuyama Y . The number of events per confounder for valid estimation of risk difference using modified least‐squares regression. Am J Epidemiol. 2018;187(11):2481–2490. 10.1093/aje/kwy158 30060121

[18] Cheung YB . A modified least‐squares regression approach to the estimation of risk difference. Am J Epidemiol. 2007;166(11):1337–1344. 10.1093/aje/kwm223 18000021

[19] Ota K , Nishioka D , Ito Y , Hamada E , Mori N , Nishii T , et al. Regression discontinuity of blood culture contamination rate after changing of disinfectants: retrospective observational study. Sci Rep. 2021;11(1):1–7. 10.1038/s41598-021-00498-x 34707137 PMC8551281

[20] Gowardman JR , Robertson IK , Parkes S , Rickard CM . Influence of insertion site on central venous catheter colonization and bloodstream infection rates. Intensive Care Med. 2008;34(6):1038–1045. 10.1007/s00134-008-1046-3 18317732

[21] Lorente L , Jiménez A , Iribarren JL , Jiménez JJ , Martín MM , Mora ML . The micro‐organism responsible for central venous catheter related bloodstream infection depends on catheter site. Intensive Care Med. 2006;32(9):1449–1450. 10.1007/s00134-006-0266-7 16804728

[22] Ota K , Takeda Y , Nishioka D , Oka M , Hamada E , Ota K , et al. Risk factors for contaminated blood cultures in the emergency department: a prospective cohort study. Microb Risk Anal. 2023;24:100264. 10.1016/j.mran.2023.100264

[23] Stohl S , Benenson S , Sviri S , Avidan A , Block C , Sprung CL , et al. Blood cultures at central line insertion in the intensive care unit: comparison with peripheral venipuncture. J Clin Microbiol. 2011;49(7):2398–2403. 10.1128/JCM.02546-10 21525219 PMC3147831

[24] Ota K . Contamination of blood cultures from indwelling arterial catheters in critically ill patients: alternative blood culture sampling? Chest. 2023;164(1):11–12. 10.1016/j.chest.2023.02.030 37423689

[25] Marschall J , Mermel LA , Fakih M , Hadaway L , Kallen A , O'Grady NP , et al. Strategies to prevent central line–associated bloodstream infections in acute care hospitals: 2014 update. Infect Control Hosp Epidemiol. 2014;35(7):753–771. 10.1086/676533 24915204

[26] Nakayama I , Izawa J , Gibo K , Murakami S , Akiyama T , Kotani Y , et al. Contamination of blood cultures from arterial catheters and peripheral venipuncture in critically ill patients: a prospective multicenter diagnostic study. Chest. 2023;164(1):90–100. 10.1016/j.chest.2023.01.030 36731787

[27] Self WH , Speroff T , McNaughton CD , Wright PW , Miller G , Johnson JG , et al. Blood culture collection through peripheral intravenous catheters increases the risk of specimen contamination among adult emergency department patients. Infect Control Hosp Epidemiol. 2012;33(5):524–526. 10.1086/665319 22476282 PMC5242221

[28] Ombelet S , Barbé B , Affolabi D , Ronat JB , Lompo P , Lunguya O , et al. Best practices of blood cultures in low‐ and middle‐income countries. Front Med. 2019;6:131. 10.3389/fmed.2019.00131 PMC6591475 31275940

[29] Benjamin RJ , Dy B , Warren R , Lischka M , Eder AF . Skin disinfection with a single‐step 2% chlorhexidine swab is more effective than a two‐step povidone‐iodine method in preventing bacterial contamination of apheresis platelets. Transfusion. 2011;51(3):531–538. 10.1111/j.1537-2995.2010.02868.x 20860675

[30] Yasuda H , Sanui M , Abe T , Shime N , Komuro T , Hatakeyama J , et al. Comparison of the efficacy of three topical antiseptic solutions for the prevention of catheter colonization: a multicenter randomized controlled study. Crit Care. 2017;21(1):320. 10.1186/s13054-017-1890-z 29268759 PMC5740719

[31] Benenson RS , Kepner AM , Pyle DN , Cavanaugh S . Selective use of blood cultures in emergency department pneumonia patients. J Emerg Med. 2007;33(1):1–8. 10.1016/j.jemermed.2006.12.034 17630066

[32] Coburn B , Morris AM , Tomlinson G , Detsky AS . Does this adult patient with suspected bacteremia require blood cultures? JAMA. 2012;308(5):502–511. 10.1001/jama.2012.8262 22851117

[33] Long B , Koyfman A . Best clinical practice: blood culture utility in the emergency department. J Emerg Med. 2016;51(5):529–539. 10.1016/j.jemermed.2016.07.003 27639424

[34] Spoorenberg V , Prins JM , Opmeer BC , de Reijke TM , Hulscher MEJL , Geerlings SE . The additional value of blood cultures in patients with complicated urinary tract infections. Clin Microbiol Infect. 2014;20(8):O476–O479. 10.1111/1469-0691.12491 24304179

[35] Ota K , Oba K , Ito Y , Cheng J , Ota K , Takasu A . Focused Assessment with Sonography for Trauma (FAST) training for first‐year resident physicians at a university hospital in Japan: a longitudinal, observational study. SAGE Open Med. 2021;9:2–7. 10.1177/20503121211044367 PMC8422809 34504709

[36] Roth A , Wiklund AE , Pålsson AS , Melander EZ , Wullt M , Cronqvist J , et al. Reducing blood culture contamination by a simple informational intervention. J Clin Microbiol. 2010;48(12):4552–4558. 10.1128/JCM.00877-10 20881178 PMC3008442

[37] Gibb AP , Hill B , Chorel B , Brant R . Reduction in blood culture contamination rate by feedback to phlebotomists. Arch Pathol Lab Med. 1997;121(5):503–507. http://www.ncbi.nlm.nih.gov/pubmed/9167605 9167605

