# Supplementary material for: Sites of blood collection and topical disinfectants associated with contaminated cultures: An ambidirectional cohort study
Source: J Gen Fam Med. 2023 Dec 17;25(1):45–52. doi: 10.1002/jgf2.667 (PMC10792313; doi:10.1002/jgf2.667)
Supplement: Supplementary file 1 — Table S1. [file JGF2-25-45-s001.docx]

Supplemental Table 1. Multivariate analysis using modified least-squares regression.

|  | Risk difference | 95% confidence interval | | | P |
| --- | --- | --- | --- | --- | --- |
| **Explanatory Variable** | |  |  |  |  |
| Disinfectants and blood sampling sites | | |  |  |  |
| ACHX and Venous | Reference |  |  |  |  |
| ACHX and Other | -0.9 | -4 | - | 2.2 | 0.566 |
| ACHX and Femoral | 0.4 | -3.1 | - | 4 | 0.815 |
| ACHX and CV catheter | -2.6 | -6.4 | - | 1.2 | 0.182 |
| ACHX and Venous catheter | -2.6 | -5.1 | - | -0.1 | 0.045 |
| PVI and Other | 7.5 | -6.1 | - | 21.2 | 0.28 |
| PVI and Femoral | 25.9 | 20.7 | - | 31.2 | <0.001 |
| PVI and Venous | 2.4 | -1.1 | - | 6 | 0.18 |
| PVI and CV catheter | 38.1 | 19.3 | - | 56.9 | <0.001 |
| PVI and Venous catheter | 1 | -6.5 | - | 8.4 | 0.798 |
| Other types and Other | -2.8 | -6.2 | - | 0.6 | 0.108 |
| Other types and Femoral | 3.8 | -9.7 | - | 17.4 | 0.578 |
| Other types and Venous | -2.3 | -5.5 | - | 0.9 | 0.159 |
| Other types and CV catheter | -4.5 | -8.4 | - | -0.6 | 0.024 |
| Other types and Venous catheter | -1 | -4.9 | - | 2.9 | 0.617 |
| **Covariates** |  |  |  |  |  |
| Male (reference female) | 0.1 | -3.8 | - | 4 | 0.959 |
| Age (per 10 years) | 1.1 | 0 | - | 2.2 | 0.041 |
| Second- year residents (reference First-year residents) | -1.4 | -6.9 | - | 4.1 | 0.624 |
| Physicians experienced less than or equal to10 years (reference First-year residents) | 0.6 | -4 | - | 5.3 | 0.787 |
| Physicians experienced more than 10 years (reference First-year residents) | -1.9 | -9.6 | - | 5.9 | 0.633 |

ACHX, 1.0% alcohol/chlorhexidine gluconate; CI, confidence interval; CV catheter, blood culture sample from newly inserted central venous catheter; Femoral, blood culture sample from femoral artery or vein; Other, blood culture sample from newly inserted arterial catheter and implanted port; Other types, alcohol and benzalkonium; PVI, 10% aqueous povidone-iodine; Venous, venipuncture without catheter insertion; Venous catheter, blood culture sample from newly inserted venous catheter.

First-year residents mean first-year trainees (postgraduate year 1 (PGY1)).

Second- year residents mean second-year trainees (postgraduate year 2 (PGY2)).

Physicians experienced less than or equal to 10 years mean that physicians who had finished their postgraduate clinical training for 2 years, and their experience was more than 2 years and less than or equal to 10 years.

Physicians experienced more than 10 years mean that physicians who had finished their residency program, and their experience was more than 10 yeans.
